# Challenges in estimating percent inclusion of alternatively spliced junctions from RNA-seq data

**DOI:** 10.1186/1471-2105-13-S6-S11

**Published:** 2012-04-19

**Authors:** Boyko Kakaradov, Hui Yuan Xiong, Leo J Lee, Nebojsa Jojic, Brendan J Frey

**Affiliations:** 1 Department of Electrical and Computer Engineering, University of Toronto, ON, Canada; 2 Microsoft Research, Redmond, WA, USA

## Abstract

Transcript quantification is a long-standing problem in genomics and estimating the relative abundance of alternatively-spliced isoforms from the same transcript is an important special case. Both problems have recently been illuminated by high-throughput RNA sequencing experiments which are quickly generating large amounts of data. However, much of the signal present in this data is corrupted or obscured by biases resulting in non-uniform and non-proportional representation of sequences from different transcripts. Many existing analyses attempt to deal with these and other biases with various task-specific approaches, which makes direct comparison between them difficult. However, two popular tools for isoform quantification, MISO and Cufflinks, have adopted a general probabilistic framework to model and mitigate these biases in a more general fashion. These advances motivate the need to investigate the effects of RNA-seq biases on the accuracy of different approaches for isoform quantification. We conduct the investigation by building models of increasing sophistication to account for noise introduced by the biases and compare their accuracy to the established approaches.

We focus on methods that estimate the expression of alternatively-spliced isoforms with the percent-spliced-in (PSI) metric for each exon skipping event. To improve their estimates, many methods use evidence from RNA-seq reads that align to exon bodies. However, the methods we propose focus on reads that span only exon-exon junctions. As a result, our approaches are simpler and less sensitive to exon definitions than existing methods, which enables us to distinguish their strengths and weaknesses more easily. We present several probabilistic models of of position-specific read counts with increasing complexity and compare them to each other and to the current state-of-the-art methods in isoform quantification, MISO and Cufflinks. On a validation set with RT-PCR measurements for 26 cassette events, some of our methods are more accurate and some are significantly more consistent than these two popular tools. This comparison demonstrates the challenges in estimating the percent inclusion of alternatively spliced junctions and illuminates the tradeoffs between different approaches.

## Introduction

Determining the relative abundance of gene transcripts in a cell - whether in relation to each other or in relation to corresponding transcripts in other cells - is an important and long-standing problem in genomics. Since introduction of RNA-seq, a high-throughput experimental method of measuring the RNA content of a sample by reverse-transcribing it and sequencing the resultant cDNA, this problem has been illuminated by vast amounts of data and by many methods for elucidating transcript abundance [1]. Current collections of RNA-seq data are rapidly growing in multiple dimensions such as species, tissues, and conditions [2].

This data deluge necessitates more sophisticated and accurate analysis methods, which in turn create an opportunity to gain deeper insights into the role and regulation of transcript abundance in important developmental and disease processes. Undoubtedly, one important research area that can benefit from these advances is the study of RNA splicing, an essential cellular process that effectively increases the phenotypic complexity of eukaryotic organisms without necessitating an increase in their genetic complexity. Accurate measurements of the expression levels for isoforms from a large number of genes are especially useful for research into the molecular mechanisms that regulate alternative splicing in different tissues. For example, the recent advances in the RNA splicing code that determines the relative abundance of alternatively spliced isoforms [3] was made possible by high-throughput microarray technology. In principle, RNA-seq can lead to much richer datasets at a fraction of the cost. Thus RNA-seq technology can lead to significant new breakthroughts, as the code quality achieved by [3] leaves a lot of room for improvement. The focus of this paper - estimation of the percent inclusion of alternatively-spliced exons from RNA-seq data - is a step toward a more accurate interpretation of the natural splicing code. This problem is complicated by several sources of bias in short read counts including those due to the cDNA fragmentation and primer amplification steps of current RNA-seq protocols [4,5]. These biases lead to widely varying abundances for reads from different positions in the transcript. We investigate this position-specific bias further and suggest methods to mitigate it.

Specifically, we restrict our interest only to exon-skipping events [6,7]. The numerical quantity which captures relevant information for these events is termed percent-spliced-in (PSI). For each exon-skipping event, PSI is defined as the expression of isogorms containing the alternatively spliced exon (i.e. those containing a given cassette exon and its flanking constitutive exons) as a fraction of the total expression for both alternatively and constitutively spliced isoforms (i.e. those containing the flanking exons only) which is reported in percent. Accurate estimation of PSI is not only desirable on its own, but it can also be used to improve the resolution of differential splicing and thus improve the predictive power of the splicing code [3].

There are several recent tools for estimating relative abundance of isoforms, which deal with position-specific biases in different ways [5,7-9]. MISO can directly estimate PSI specifically for exon-skipping events [7], while most others estimate the expression of whole isoforms from which a PSI value may be derived. This makes MISO the natural point of reference for our comparisons, but we also include Cufflinks [5] in the comparisons because of its popularity and explicit modeling of fragmentation and amplification biases. However, for the task of estimating PSI, Cufflinks' focus on multi-exon isoforms appears to be detrimental, as we show in the Results section.

Our pursuit of robust estimates for PSI necessitates an appropriate measure of the uncertainty for these estimates. This additional necessity is crucial for the task of deciphering the natural RNA splicing code. Linking noisy RNA-seq read counts with the sequence determinants of RNA splicing is a hard task that requires good measurement of splicing levels even in case of transcripts with minimal coverage. For this task it is just as important to quantify the range of possible PSI values supported by the RNA-seq data, given that the position-specific bias can dramatically influence these estimates. We start by framing the classic IID sampling assumption as a Poisson model and modify it to mitigate the effect of position-specific biases. This leads to three methods of increasing complexity. We evaluate our models in terms of their accuracy and consistency. We compare our methods' accuracy to each other and to existing approaches of estimating PSI with respect to a reference set of 26 RT-PCR measurements from a human cell line. As we discussed above, we are interested in developing algorithms that provide robust estimates: A handful of highly biased positions in the transcript, from which a much larger number of reads is obtained simply due to fragmentation bias, should not unduly influence the estimate of PSI. Our results show a moderate increase in accuracy and a significant increase in consistency of our methods over the current state of the art methods for quantifying of alternative splicing events.

## Methods

### RNA-seq data

RNA-seq data was generated from a HeLa cell line by the Blencowe Lab at the University of Toronto [10]. The protocol consisted of polyA-selected RNA extraction, random hexamer primed reverse transcription, cDNA fragmentation (with mean insert size of 220nt), and 50nt paired-end sequencing by Illumina GA. This dataset is publicly available on the NCBI Gene Expression Omnibus with accession number GSE26463. 305 million RNA-seq reads were sequenced and mapped to the reference human genome (NCBI build37, UCSC hg19) using TopHat, which is capable of reporting split-read alignments across splice junctions [11]. TopHat produced error-free alignments for 66 million reads (about 22% of the total). For each exon-exon junction, the reads that overlapped it by at least 8nt were selected and their positions were noted. Positions that contained reads mapping elsewhere were excluded. The number of 3' fragment ends (i.e. reads starts) around the junction was tabulated into a profile of read hits for each junction. This profile of read start counts is also called a read cover, in contrast to the more popular read coverage.

**Figure 1:**
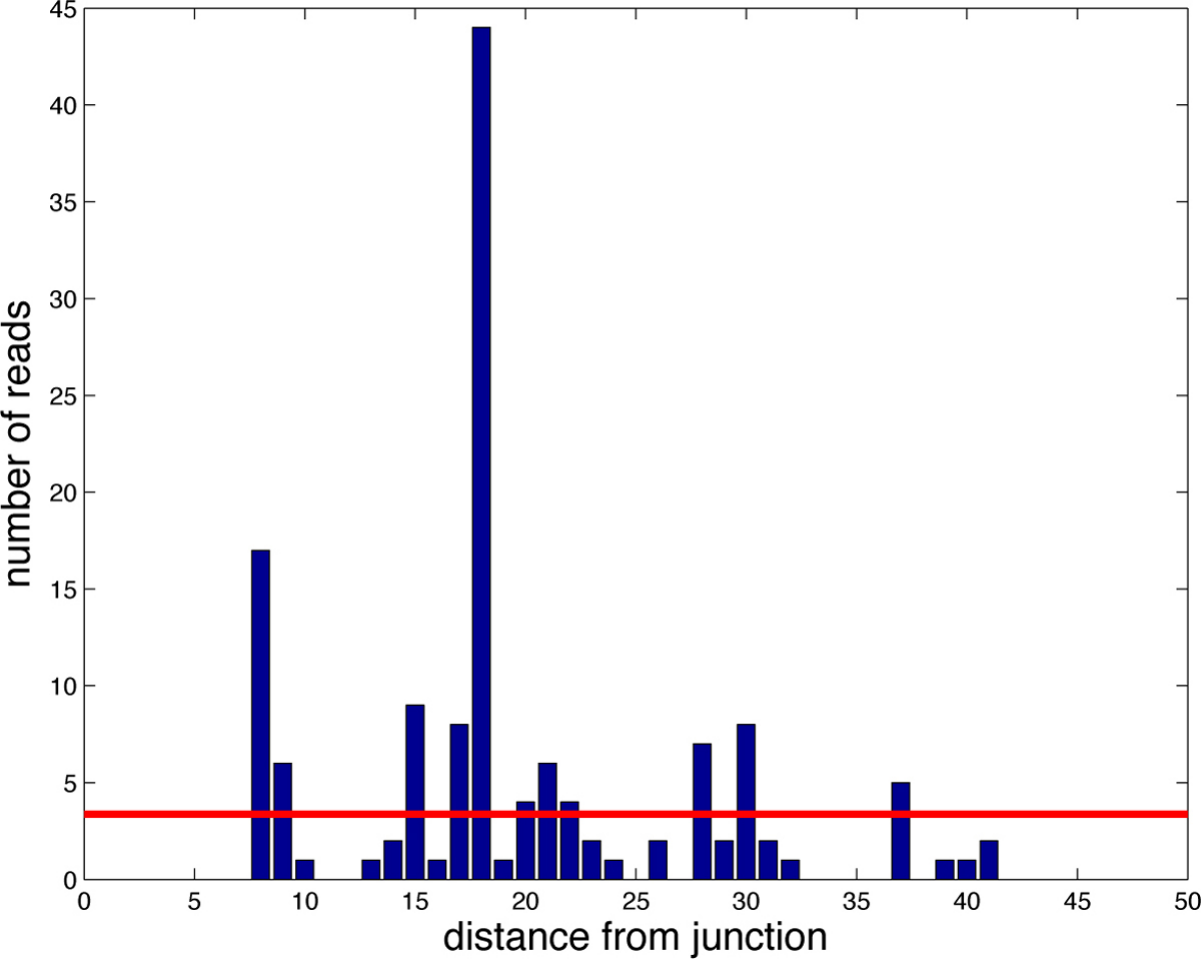
**Read cover of sample junction**. A read cover profile shows the number of read alignments (y-axis) that start at a particular distance (x-axis) from the splice junction. This histogram is a typical example of the 50nt neighborhood around a highly expressed constitutive junction. This example exhibits two types of read mapping bias: sparse coverage (empty positions) and read-stacks (tall blue bars). The horizontal line (in red) *α *= 3.4 marks the average expression of the junction determined by the Native model.

Figure 1 illustrates the actual cover profile for a representative constitutive (i.e. exclusion) junction with a relatively high total number of reads. Position-dependent biases in the read cover lead to positions with zero reads, as well as positions with many mode reads than are expected based on other positions. These two situations are sometimes treated differently, but they are essentially due to the same cause: position-dependent effects. Note that these position-dependent effects are present in the majority of junctions regardless of their underlying expression. Another source of error is mis-matched reads but, in this work, we deliberately used only error-free alignments (as opposed to the common practice of allowing a small number of mismatches) in order to differentiate the positional biases from mismatch noise. When estimating PSI, the individual read covers for each pair of alternative junctions that flank an alternative exon can be tabulated into a joint inclusion junction cover using half-counts at each position. This is common practice for analyses of alternative splicing as it is assumed that the increased sample size results in better estimates of expression. However, we note that averaging the read covers for the two alternative junctions is not appropriate when the constitutive annotation of the two flanking exons is in question, and this approach does not significantly reduce the harsh effects of positional biases.

The existing tools for isoform quantification, MISO and Cufflinks were provided with the entire alignment, not just the reads mapping to junctions. MISO (version 0.2) and Cufflinks (version 1.2) were run with default parameters except for the paired-end read insert size and the number of samples from the appropriate posterior, which were set to 220 and 10000, respectively.

### Native model

The first model we study makes the simplifying assumption that reads are sampled independently and identically distributed (IID) from the expressed isoforms. We refer to it as the "Native" model, because its key component, the Poisson arrival process, is a natural model for IID read coverage. This "Native" model has worked sufficiently well in the past for analysis in many respectable DNA and RNA sequencing studies [2].

Many simple models of RNA-seq data assume, either explicitly or implicitly, that reads are sampled uniformly along the length of a transcript [1,12]. However, actual RNA-seq data do not follow this assumption because of multiple sequence- and position-specific biases inherent in the cDNA library preparation and sequencing [4,5,7,13]. Still, we might expect this assumption to hold for sufficiently short regions on a transcript, such as the neighborhood around an exon-exon junction. In this case, the number of read starts *x*_*p *_mapping to each position *p *near the junction should follow a Poisson distribution whose mean is estimated by $\tilde{\alpha }=\frac{1}{P}\sum_{p}x_{p}$ where the region of interest spans positions {1, 2, ... *P*}. The mean and matching variance *α *will estimate both the overall expression for that junction and the model's uncertainty in that expression. Unfortunately, reads are not distributed uniformly, even along short regions with sufficient coverage. As shown on Figure 1, the read counts covering the region within 50nt of a representative constitutive junction are highly variable and non-uniform. The corresponding cover for the two alternative junctions (not shown) contains about twice as many read counts in total, but they are split over two neighborhoods of 50nt. In general, RNA-seq data deviates from the Native Poisson model in two ways:

• the high sparsity of the data (~ 80% of positions have no reads starting at them) causes$\tilde{\alpha }$, the average cover for the region, to underestimate the expected abundance *α.*

• the variance of the non-zero elements *x*_*p *_> 0 is three times larger than that dictated by the Native model.

Note that the Poisson model describes the likelihood *P*(*x*_*p *_| *α*) of observing a particular read cover profile *x*_*p *_given the unknown expression *α*. However, we are interested in the posterior probability *P*(*α *| *x*_*p*_) of the hidden expression given the observed data. This posterior can be obtained from the likelihood of the observed data and the prior over the expression through the classic Bayes' Rule:

(1) $$P\left(\alpha |x\right)=\frac{P\left(x|\alpha \right)*P\left(\alpha \right)}{P\left(x\right)}$$

Once we have distributions over the expected expression for both the alternative (a.k.a. inclusion) and the constitutive (a.k.a exclusion) junctions, α^*i *^and *α*^*e *^respectively, we combine them to produce the posterior over the PSI estimate of this model $P\left(\Psi_{\text{Native}}|x_{p}^{i},x_{p}^{e}\right)$ given the observed read counts over the inclusion ($x_{p}^{i}$) and exclusion ($x_{p}^{e}$) junctions, respectively. There is no closed-form expression for this distribution, but we can estimate it with the ratios of samples from the inclusion and exclusion posteriors:

(2) $$P(\Psi_{\text{Native}}|x_{p}^{i},x_{p}^{e})\propto \sum_{\underset{\frac{\alpha^{i}}{\alpha^{i}+\alpha^{e}}=\Psi_{\text{Native}}}{\alpha^{i},\alpha^{e}:}}P(\alpha^{i}|x_{p}^{i})*P(\alpha^{e}|x_{p}^{e})$$

### Gaussian model

In order to alleviate the shortcomings of the Native model, we propose two simple modifications which result in a new Gaussian model that is more robust to the position-specific biases present in RNA-seq data. To deal with the sparse cover and its effect on the expected expression, *α*, we dismiss all unmappable positions, i.e. those positions which coincide with the start of reads that map elsewhere in the reference genome or transcriptome. This leaves only the set of position indexes *Q *which coincide with the hits of only uniquely-mappable reads. Therefore, the normalized expression of a junction is $\gamma =\frac{1}{\left|Q\right|}\sum_{q\in Q}x_{q}+\frac{1}{p}$ where we have added the pseudo-count $\frac{1}{P}$ in order to avoid dividing by zero for junctions which have no uniquely-mappable reads, e.g. those that come from homologous regions of the genome.

To deal with the high variance at positions with non-zero read count, we approximate the PSI ratio of normalized junction expressions with a Gaussian distribution. Unlike the Poisson distribution whose mean and variance are identical by definition, the link between the mean and variance of this Gaussian approximation can be relaxed in order to make the model more robust. The mean *μ *is estimated by the ratio of the normalized read counts for the inclusion and exclusion junctions (*γ*^*i *^and *γ*^*e*^, respectively). The standard deviation *σ *is proportional to the geometric mean of *μ *and its complement 1 - *μ*. The variance *σ*^*2 *^is normalized by the total number of uniquely mappable reads in the alternative and constitutive junction Γ = *γ*^*i*^|*Q*^*i*^| + *γ*^*e*^|*Q*^*e*^|, where |*Q*^*i*^| is the number of uniquely-mappable positions for the inclusion junction, and |*Q*^*i*^| is that for the exclusion junction. Finally, the variance is lower-bounded by an arbitrary threshold in order to avoid over-fitting the noisy RNA-seq data:

(3) $$\tilde{\mu }=\frac{\gamma^{i}}{\gamma^{i}+\gamma^{e}}\;\;{\tilde{\sigma }}^{2}=\text{max}\left[0.01,\frac{\tilde{\mu }\left(1-\tilde{\mu }\right)}{\Gamma }\right]$$

This approximation allows us to skip the Bayesian procedure and sampling approximation required by the Native model, since we can directly specify the posterior distribution of our estimate for PSI given the read counts around a junction: $P\left(\Psi_{\text{Gaussian}}|x_{p^{\prime }}\right)\sim N\left(\tilde{\mu },{\tilde{\sigma }}^{2}\right)$.

### Bootstrap technique

To robustly estimate PSI without explicitly modeling sequence and position dependent bias, we propose a method based on randomly resampling the observed data. This method computes the degree of uncertainty in PSI by estimating the consistency within the observed dataset. It belongs to a general class of statistical methods called bootstraping that have been successfully used to model complex and unknown distributions [14].

The bootstrap can be used to assess the uncertainty in the PSI estimates produced by any method that takes position-dependent read counts as input. Here, we use a Poisson model. We assume that there are *P *mappable junction positions for each exon skipping event. We observe $x_{p}^{i}$ inclusion reads and $x_{p}^{e}$ exclusion reads for each position *p *= {1, 2, ... *P*}. To estimate PSI from such a dataset, a simple approach assumes that for every position, $x_{p}^{i}$ and $x_{p}^{e}$ are generated by a Poisson distribution with real-valued underlying abundances *β*^*i *^and *β*^*j *^respectively. A Poisson distribution is used to model the process of how RNA-seq reads in each position arise from the true abundance of isoforms in the biological sample. Because of the IID assumption, the maxmimum likelihood (ML) estimator of *β *is simply the sum of the observed reads. Instead of simply using the ML estimator, we take a Bayesian approach where we assume an improper prior for *P*(*β*) = 1 for the abundances of both inclusion and exclusion variants. The posterior of *β *is a Gamma distribution with a shape parameter equal to 1:

(4) P(β)=1;

(5) $$P\left(\vec{x}|\beta \right)=\underset{k}{\prod }P\left(x_{p}|\beta \right);$$

(6) $$P(x_{p}|\beta )=\text{Poission(}x|\beta );$$

(7) $$=\frac{\beta^{x_{p}}}{x_{p}!}e^{-\beta };$$

(8) $$P\left(\beta |\vec{x}\right)\propto P\left(\beta \right)P\left(\vec{x}|\beta \right);$$

(9) $$\propto \frac{\beta^{\sum_{p}x_{p}}}{\left(\sum_{p}x_{p}\right)!}e^{-\beta };$$

(10) $$P\left(\beta |\vec{x}\right)=\text{Gamma}\left(1,1+\underset{p}{\sum }x_{p}\right),$$

where Gamma(*θ*, *k*) denote the real valued Gamma distribution with scale parameter *θ *and shape parameter *k*. In this application, the shape parameter is one plus the sum of the reads across positions. The Gamma random variable in the above equation incorporates our belief of likely values of isoform abundances (*β*) given the observed reads, with the IID assumption for read generation across positions. However, the IID assumption described above is highly incorrect, because of position-dependent effects introduced by RNA-seq technologies. We use the bootstrap to assess the uncertainty induced by these effects as follows. Instead of summing over the reads at all positions, we generate a sample of *P *positions with replacement from the observed data and then sum the reads at those positions to produce an estimate of *β *as described above.

The above procedure is repeated to generate a distribution of *β *estimates, which can be used to form a distribution of PSI. In our approach, one million *β*^*i *^and *β*^*e *^are generated with which one million samples of Ψ_bootstrap _are produced.

### Robust mixture model

We propose a robust mixture model of read counts that span alternatively-spliced junctions from exon skipping events. The mixture has three components:

1. A zero-cover component to explain the empty positions arising from sparse fragmentation bias.

2. A noise component to capture the read stacks arising from the other type of positional bias.

3. A Poisson component to capture the remaining signal in the read cover.

Formulating a mixture model allows us to explicitly capture each of the two types of bias alongside the underlying signal in RNA-seq data.

For each cassette splicing event, our model links the hidden expression counts λ^*i *^and λ^*e*^, for the inclusion and exclusion junctions, to the unknown PSI and coverage values: $\Psi_{\lambda }\in \mathbb{Q}$ and *C *∈ ℤ, and to the observed read counts: $x_{p}^{i}\in \mathbb{Z}$ and $x_{p}^{i}\in \mathbb{Z}$ where *p *∈ {1, 2, ..., *P*} are positions in the neighborhood of each junction. As before, Ψ_λ_, *C*, and *λ *are linked by a deterministic relationship:

(11) $$\Psi_{\lambda }=\frac{\lambda^{i}}{C}\;\text{where}\;\;C=\lambda^{i}+\lambda^{e}$$

**Figure 2:**
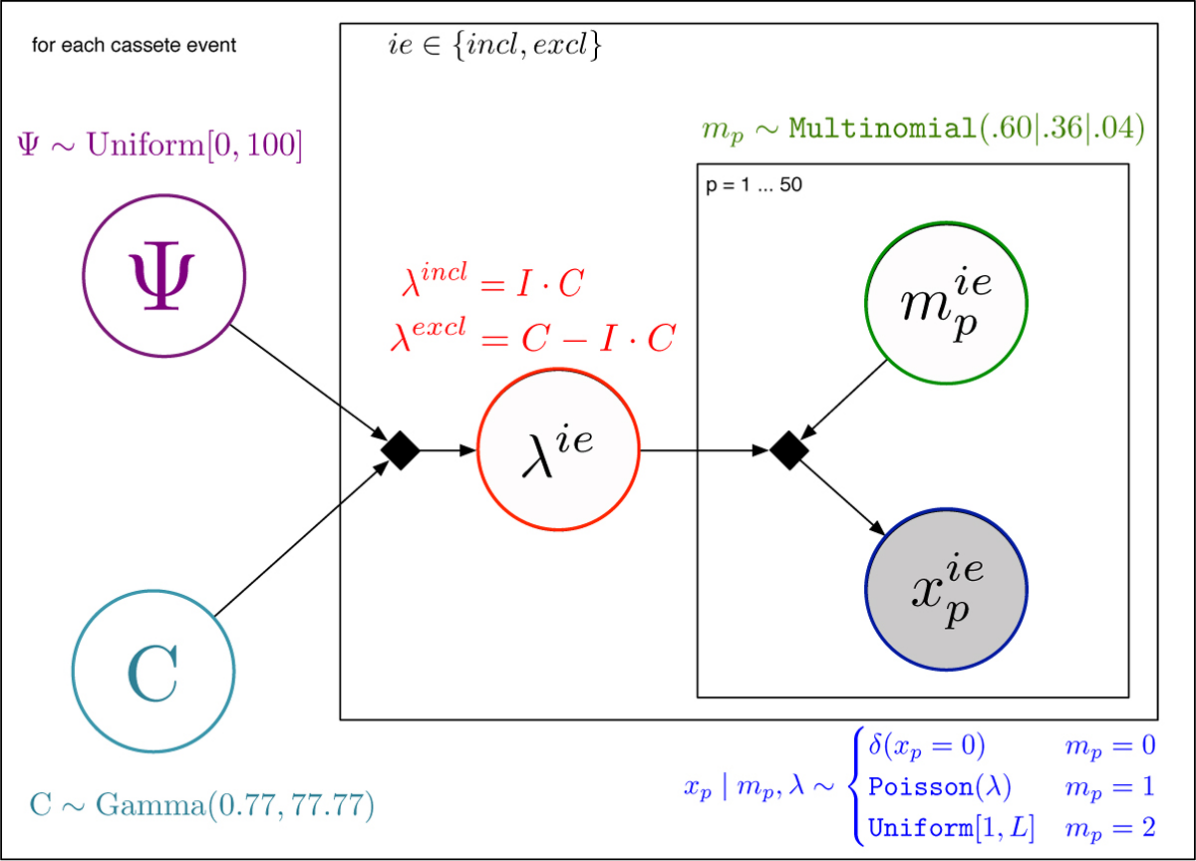
**Plate model for Robust Mixture**. Our Mixture Model for robust estimation of PSI and coverage of cassette junctions from RNA-seq data. Only the read counts at each position (shaded *x*_*p*_) are observed. The mixture components (*m*_*p*_), robust expression estimates for each junction (λ^*ie*^), and the overall cover (*C*) and percent-spliced-in (Ψ) are inferred by the model.

Figure 2 shows the plate diagram for the Robust Mixture model. Its priors and factors are described in the following sections. The the priors and factors combine via Bayes' Rule (already described in Equation (1)) to give the posterior distribution over the hidden variables and mixture weights of this model.

#### Priors

• PSI: Ψ_λ _~ Uniform[0, 1]

  even though the empirical distribution is closer to a convex Beta distribution with preference for extreme values of Ψ_λ_, we use the least informative prior in order to gain the most information about this hidden variable of interest [7].

• Cover: *C *~ Gamma(*θ*, *k*)

   with scale parameter *θ *= 77.77 and shape parameter *k *= 0.77 estimated from *C*'s empirical distribution.

• Expression: A complex prior on λ^*i *^and λ^*e *^is induced by the priors on *Ψ*_λ _and *C *through the relation in equation (11). We impose no further restriction on the distribution of these hidden variables.

• Mixture: The weights of the three mixture components represent the relative strengths of the signal and the two noise models. The observed sparsity of RNA-seq data ( where 80% of junction-neighboring positions have no read alignments starting from them) is an upper bound on the true sparsity because we expect to see zero-cover positions in junctions with very low expression. Therefore we chose 60% sparsity as a reasonable compromise. Likewise, the observed read-stack outlier rates for the Illumina platform is a lower bound on the actual fraction of outlier reads (3% of all junction-adjacent positions have a read count that is 10× higher than the simple average).

(12) $$p_{0}\left(m_{p}\right)=\left\{\begin{array}{cc} 0.60 & \text{Zero}\;\text{Cover }\left(m_{p}=0\right)\\ 0.36 & \text{Poisson}\;\text{Model}\;\left(m_{p}=1\right)\\ 0.04 & \text{Read}\;\text{Stacks }\left(m_{p}=2\right) \end{array}\right)$$

#### Factors

• Deterministic: λ^*i*^, λ^*e *^~ *δ*(λ^*i *^= *Ψ*_λ _* *C*)*δ*(*λ*^*e *^= *C *- λ^*i*^)

• Multinomial: *m*_*p *_~ Multinomial(*c*_*z*_, *c*_*p*_, *c*_*s*_)

  This factor allows our model to learn the actual mixture weights for each of the components from the observed data.

• Mixture: We use a mixture factor in order to capture each of the two biases and the actual signal in separate components. The choice for each component is motivated by the form of the signal or noise it is designed to capture.

(13) $$x_{p}|m_{p,}\lambda \sim \left\{\begin{array}{cc} \delta \left(x_{p}=0\right) & \text{Sparsity }\left(m_{p}=0\right)\\ \text{Poisson }\left(\lambda \right) & \text{Signal }\left(m_{p}=1\right)\\ \text{Uniform }\left[1,L\right] & \text{Noise }\left(m_{p}=2\right) \end{array}\right)$$

### Practical considerations

**Figure 3:**
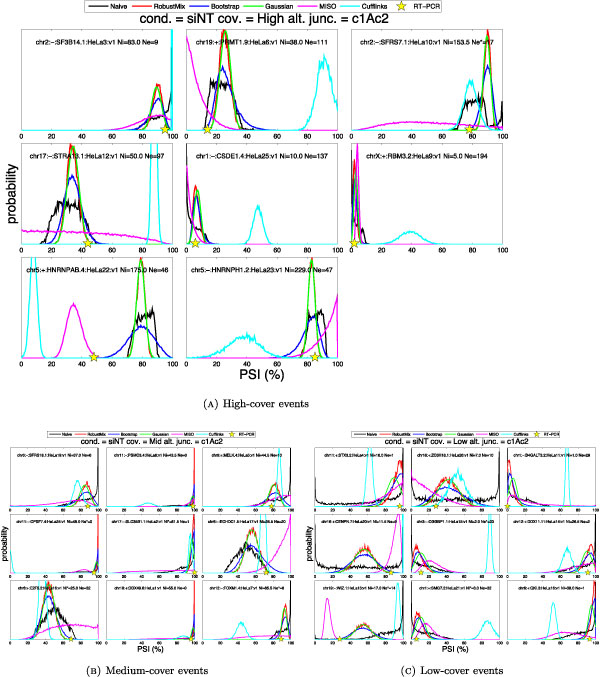
**Comparison of PSI estimates**. Comparison of PSI estimators of different methods for (a) high- (b)medium- and (c) low-cover junctions in a reference RT-PCR study. Each method's estimated distribution over PSI is shown in different color, and the target PSI value is shown as a yellow star on the x-axis. Methods which commit the most of their distribution mass near the star have the most accurate estimates. The text inside each plot identifies a cassette event and gives the raw number of reads mapping to the constitutive (Ne) and the average of the alternative junctions (Ni). This figure is best viewed in color.

Performing inference in the Native and Robust Mixture models described above is intractable due to the complex partition function that normalizes the posterior distribution *P*(Ψ|*x*_*p*_). To compute the posterior, we could use advanced approximate inference methods such as Expectation Maximization used by IsoEM [8], Markov Chain Monte Carlo used by MISO [7], and combinatorial optimization used by Cufflinks [5,12]. However, we note that discretizing the values of their parameters allows us to approximate the partition function and directly calculate the posterior distribution over the discretized PSI values: Ψ_*α *_and Ψ_λ _respectively. In contrast, the Gaussian and bootstrap models give a posterior over Ψ_*γ *_directly, either in a closed form expression or in the form of samples from a provably exact distribution. Figure 3 shows that the resulting posterior distributions for all PSI estimators are well-formed, especially for junctions with sufficiently high read cover, and gives support for the viability of our discretization scheme for junctions of medium or even low read cover. Finally, performing inference with discretized parameters takes considerably less time at a minimal loss of precision. This allows our methods to analyze an entire pre-aligned RNA-seq dataset in the manner of a few minutes, while other methods take tens of hours or even days on the same task, while other methods take hours on the same task.

## Results and discussion

### Accurate estimation of PSI

In order to evaluate the accuracy of our models and compare it to that of the existing methods, we selected a validation set of 26 cassette exons with reference PSI values derived from RT-PCR experiments in HeLa cells [10]. The 26 events include 11 high-expression events with between 10 and 20 read starts per position, 8 medium-expression events with about 1 read start per position, and 7 low-expression events with 10 or fewer reads total across all 50 positions (≤ 0.2 read starts per position). Figure 3 compares the posterior distributions over PSI inferred by six different methods: our four methods described in the Methods section, and two popular tools for isoform quantification, MISO and Cufflinks. All tools shared the same input, but were able to extract varying amount of information from it. The shared TopHat alignment file included the mapping of reads to a reference set constructed only from the constitutive and alternative exons of the 26 cassette events. Our tools were able to use only the reads mapping across junctions, while MISO and Cufflinks was free to use the entire set of alignments. Furthermore, our methods did not benefit from the paired-end dependencies between the reads, while both MISO and Cufflinks were able to do so. To be fair, we note that Cufflinks is designed for whole-transcript quantification. Thus, we did not expect it to be competitive with the other methods on a highly restricted reference set consisting of only three exons per alternative splicing event

While limited, this comparison clearly shows that no particular method outperforms the others on every event. However, it does suggest that our methods are more accurate, especially when they agree with each other. We investigate the consistency of our methods in a later part of the Results section. Unfortunately there is no canonical way to measure the error between a distribution estimate and a point target. However, we modify three existing distance metrics between distributions and propose a new metric which allow us to compute the overall performance of the six methods on all 26 events. Given a PDF distribution of PSI estimates *P*(*x*) and a target value *ψ *described by discretized Gaussian distribution *Q*_*ψ*_(*x*) centered at the point target, *ψ*. We used an arbitrary standard deviation *σ *= 0.05 which is comparable to the accuracy needed for downstream applications of PSI estimates. The new metric directly computes the distance between a distribution and its target.

• Variation distance, which measures the total deviation between the two distributions

(14) $$V\left(P,Q_{\psi }\right)=\underset{0\leq x\leq 1}{\sum }|P\left(x\right)-Q_{\psi }\left(x\right)|$$

• Disagreement distance between CDFs, which measures the maximum deviation. In our case, the maximum is attained at the mode of either *P *or *Q*_*ψ*_

(15) $$S\left(P,Q_{\psi }\right)=\underset{0\leq y\leq 1}{\text{max}}\underset{0\leq x\leq y}{\sum }P\left(x\right)-Q_{\psi }\left(x\right)$$

• KL divergence, which measures the asymmetric disagreement between *P *or *Q*_*ψ *_with respect to the latter

(16) $$D_{\text{KL}}\left(Q_{\psi }∥P\right)=\underset{0\leq x\leq 1}{\sum }Q_{\psi }\left(x\right)\text{log}\frac{P\left(x\right)}{Q_{\psi }\left(x\right)}$$

• Novel confidence-weighted $L_{\frac{1}{2}}$ error distance, is designed to penalize distributions that distribute weight away from the target *ψ*

(17) $$E_{\frac{1}{2}}\left(P,\psi \right)=\underset{0\leq x\leq 1}{\sum }P\left(x\right){∥x-\psi ∥}_{\frac{1}{2}}$$

Table 1 shows the overall performance of each PSI estimation method over the 26 target events according to each of these error metrics. While our most robust methods perform well on three of these metrics, it is not surprising that MISO outperforms every other method on the remaining S-metric because it always distributes its posterior mass wider than our methods. The disagreement distance, *S*(*P*, *Q*_*ψ*_) rewards this extensive hedging because it is very susceptible to sampling noise which is abundant on Figure 3. The remaining metrics are chosen to be more robust when faced with this sampling noise.

**Table 1:** Accuracy

Error	Native	Gaussian	Mixture	Bootstrap	MISO	Cufflinks
*V*	28.5	**24.1**	27.2	**24.2**	30.9	43.7
*S*	12.90	15.26	15.87	15.22	**9.87**	12.65
*D*_KL_	264	102	**94.2**	**92.0**	220	1115
*E*_1/2_	9.34	7.08	**6.62**	**6.65**	9.28	14.65

Comparison of error between different PSI estimation methods with respect to RT-PCR target. The best methods with lowest error in each row are bolded. Robust Mixture model is abbreviated to "Mixture".

### Consistent estimation of PSI

**Figure 4:**
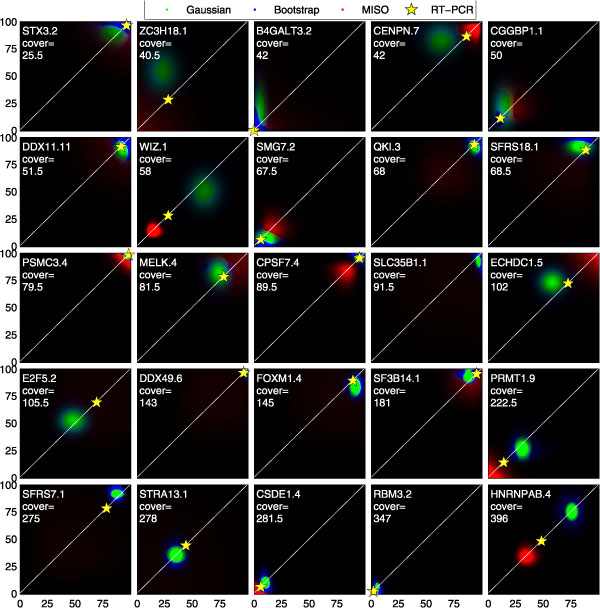
**Consistency of PSI estimates**. Constellation plot of the estimated PSI distributions from one vs. another half of the positions in each cassette event. The distribution of PSI along the x-axis, *P*_*x*_(Ψ) over the range (0-100%) is estimated from a random half of the positions and the distribution on the y-axis *Py*(Ψ) comes from the remaining half of the positions. The distributions are color-coded according to their methods. The intensity of each pixel (*x*, *y*) = (*a*, *b*) corresponds to the product of the distributions *P*_*x*_(*ψ *= *a*) * *P*_*y*_(*ψ *= *b*). In regions where the distributions for different methods overlap, the one with the higher probability is shown and the rest are suppressed. Each white diagonal marks the region of perfect agreement for both distributions. The yellow star along each diagonal is placed at the x- and y-coordinate matching the PSI value determined by RT-PCR for the event whose name and cover are printed in white font. This figure is best viewed in color.

In order to further investigate the consistency of PSI estimation methods, we performed a random sub-sampling procedure. This procedure chooses a random half of the positions around a junction and uses the subset of reads that start at those positions to obtain an unbiased estimate of the noise associated with the positional bias. A dataset with reduced set of positions is equivalent to a dataset with reduced signal-to-noise ratio. Comparing the PSI estimate of a method given each half of the positions can measure the consistency of that method. Figure 4 depicts the consistency of the most accurate methods from Table 1 with a non-standard 2D color visualization. We call a this visualization a constellation plot because of its superficial resemblance to images of deep-space galaxies.

**Figure 5:**
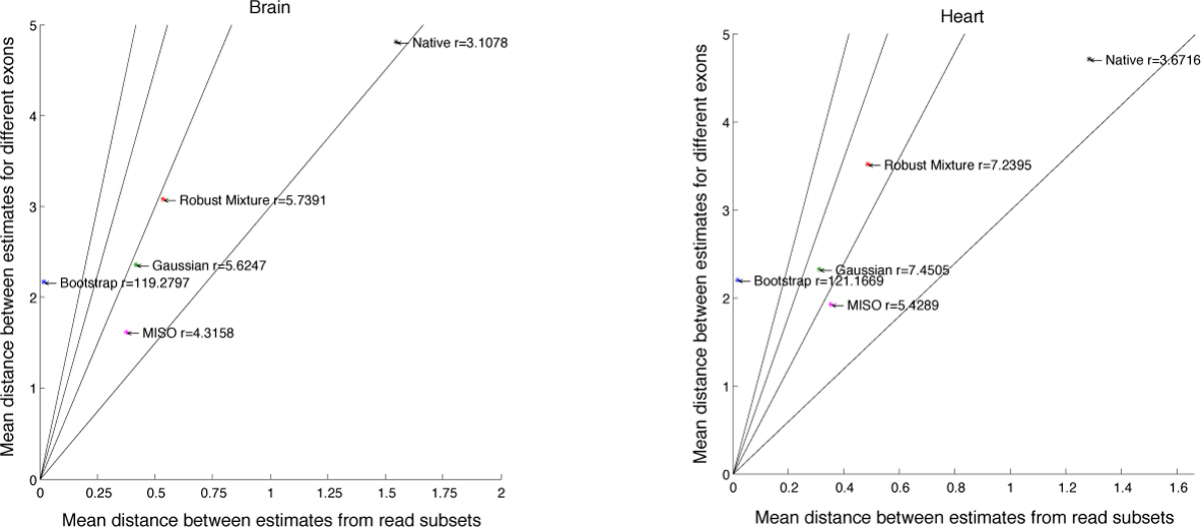
**Consistency ratios in different tissues**. Plots of the consistency ratio between inter- and intra-exon divergence in the estimated PSI distributions for five of the methods in two human tissues. The PSI estimates were generated for a random half of the positions in each junction and compared to the PSI estimate from the other half within the same exon and between different exons. More consistent methods have a higher consistency ratio.

We expect more consistent methods to produce consistently more similar estimates of PSI. For each method, we calculate the KL-divergence between its PSI estimate on a particular event to the PSI estimate on all other events. We compare the mean of all cross-event divergence to the divergence between PSI estimates from complementary halves of the same event. The former divergence we call the inter-exon distance, and the latter we call the intra-exon distance. Then, the ratio between the inter- and intra-exon distances is a measure of the method's consistency for that particular exon. More consistent methods will have a higher ratio over all events. Figure 5 compares the consistency ratios of our four methods and that of MISO using a larger dataset of over 1000 events (including the 26 validated by RT-PCR).

Consistency of the PSI estimates is especially important to the downstream uses of our methods. If only a randomly selected subset of positions are taken into account, the PSI estimate (and its uncertainty) should be very similar to the estimate that would be computed based on the complementary set of transcript positions. Thus we defined a measure of consistency of the estimator as the ratio of the average distance of the PSI distributions obtained from two different genes and the average distance from PSI distributions obtained from different position subsets of the *same *transcript. High values of this ratio indicated that using a smaller subset of the positions will not affect the estimate of PSI drastically, but that this is not achieved in a trivial way by always estimating either a high or a very low level of exon inclusion.

### Runtime and efficiency

While accuracy and consistency are the most important considerations for any approach of estimating PSI, runtime and efficiency are becoming increasingly relevant as the amount of RNA-seq data grows rapidly. Table 2 compares the runtimes of all methods on both the small validation set of 26 events and the larger set of 1051 events. To estimate the distribution over PSI values for each event, we used 10,000 samples for all methods. Sampling from the Gaussian model was direct whereas other models sampled the expression for inclusion and exclusion isoforms separately. It is not surprising that the run time of our pre-processing grows linearly with the number of RNA-seq reads, and we expect the same happens to the pre-processing subroutines of both MISO and Cufflinks. However, the estimation subroutines in the two established tools are disproportionately slower on the larger dataset than any of our simple methods, including the robust and very consistent bootstrap model.

**Table 2:** Runtime

Datasets:	Validation	High-Throughput
RNA-seq reads	66 Million	145 Million
AS events	26	1051
Cufflinks	16 min	75 min
MISO	77 min	458 min

Preprocess	4 min	11 min
Gaussian	+1 sec	+2 min
Native	+2 sec	+5 min
Mixture	+6 sec	+17 min
Bootstrap	+12 sec	+29 min

Comparison of run times between different PSI estimation methods. For our methods, we report the runtime of the shared pre-processing step separately from the PSI estimation. All tests were performed on a Dell Precision T7400 workstation with 8 cores (at 3 GHz) and 32 GB of RAM. We report wall-clock times averaged over 3 re-runs then rounded to the nearest minute (or second where appropriate).

## Conclusion

This work addressed the problem of estimating relative abundances of alternatively-spliced cassette exons from the sparse and noisy evidence in RNA-seq data. First, we investigated the raw data and reviewed known fragmentation biases resulting from current RNA-seq protocols. Next, we identified position-specific anomalies affected by these biases, and proposed a modular probabilistic framework that robustly estimates the PSI and total coverage of alternatively-spliced exon junctions. Using this foundation, we framed the classic IID read sampling assumption as a Poisson model and termed the two types of position-specific deviations in the actual data as sparse cover and read stacks. Using the established framework, we proposed three novel probabilistic methods of increasing complexity, which mitigate the effects of these two biases. We compared our methods' accuracy to each other and to existing approaches of estimating PSI with respect to a reference set of 26 RT-PCR measurements from a human cell line. Our results showed a moderate increase in accuracy and a significant increase in consistency of our methods over the current state-of-the-art for quantification of alternative splicing events. While we presented and referenced several methods for quantifying alternative splicing, our goal was not to pick a single champion that is superior to all others, but to compare the strengths and weaknesses of the various approaches. We hope that these advances will enable more sensitive downstream analyses, such as better determinants of differential splicing which can eventually lead to an improved RNA splicing code.

## Competing interests

The authors declare that they have no competing interests.

## Authors' contributions

BK identified the positional biases, developed the Robust Mixture method, performed the analyses, and drafted the manuscript. HYX developed the Bootstrap method and wrote its description. LJL pre-processed the RNA-seq data, and participated in the analysis. NJ developed the consistency ratio measure and revised the manuscript. BJF guided the study and proposed the Bootstrap method.
